# A Novel Collaboration to Reduce the Travel-Related Cost of Residency Interviewing

**DOI:** 10.5811/westjem.2017.1.33085

**Published:** 2017-02-07

**Authors:** Eric Shappell, Abra Fant, Benjamin Schnapp, Jill P. Craig, James Ahn, Christine Babcock, Michael A. Gisondi

**Affiliations:** * University of Chicago, Department of Medicine, Section of Emergency Medicine, Chicago, Illinois; † Northwestern University Feinberg School of Medicine, Department of Emergency Medicine, Chicago, Illinois; ‡ Northwestern University Feinberg School of Medicine, Department of Medical Education, Chicago, Illinois

## Abstract

**Introduction:**

Interviewing for residency is a complicated and often expensive endeavor. Literature has estimated interview costs of $4,000 to $15,000 per applicant, mostly attributable to travel and lodging. The authors sought to reduce these costs and improve the applicant interview experience by coordinating interview dates between two residency programs in Chicago, Illinois.

**Methods:**

Two emergency medicine residency programs scheduled contiguous interview dates for the 2015–2016 interview season. We used a survey to assess applicant experiences interviewing in Chicago and attitudes regarding coordinated scheduling. Data on utilization of coordinated dates were obtained from interview scheduling software. The target group for this intervention consisted of applicants from medical schools outside Illinois who completed interviews at both programs.

**Results:**

Of the 158 applicants invited to both programs, 84 (53%) responded to the survey. Scheduling data were available for all applicants. The total estimated cost savings for target applicants coordinating interview dates was $13,950. The majority of target applicants reported that this intervention increased the ease of scheduling (84%), made them less likely to cancel the interview (82%), and saved them money (71%).

**Conclusion:**

Coordinated scheduling of interview dates was associated with significant estimated cost savings and was reviewed favorably by applicants across all measures of experience. Expanding use of this practice geographically and across specialties may further reduce the cost of interviewing for applicants.

## INTRODUCTION

In 2016, the National Residency Matching Program (NRMP) Main Residency Match saw a total of 42,370 registrants; of these, 2,476 applied to at least one of the 174 categorical programs in emergency medicine (EM).^1^ The financial cost of this process is significant and increases with the number of interviews per applicant.

Data about the financial burden of the residency interview process are limited. Studies in other specialties have found total expenses ranging from $4,000 to $15,000 per applicant with travel and lodging comprising 60% and 25% of expenditures respectively.^2^–^6^ Since travel to programs outside an applicant’s region may be limited by financial burden, programs may suffer from less geographic diversity in applicant pools.

Due to significant financial burden on applicants and potential downstream effects of limited geographic diversity for training programs, we believe that interest in optimizing the interview process should be high. However, we have not found literature describing collaborative efforts among institutions to reduce the cost for applicants.

We hypothesized that we could reduce the financial burden of interviewing by offering consecutive interview dates for the EM training programs at Northwestern University (NU) and the University of Chicago (UC), both located in Chicago, Illinois. Applicants interviewing at both programs could then arrange a single trip for both interviews, thereby decreasing travel costs. We additionally hypothesized that this intervention would improve the interview experience for our applicants.

## METHODS

Interview date coordination was established between the EM residency programs at NU and UC for the 2015–2016 interview season. Anecdotal data suggested a similar applicant pool between institutions. Each program offered two back-to-back weekday interview dates with one day of overlap between programs (i.e. one program interviewed on days 1 and 2, the other program interviewed on days 2 and 3). Coordinated dates spanned October to December 2015. Both programs released initial interview offers on the same date.

We assessed this intervention using historical data from interview scheduling software, Interview Broker^®^ (The Tenth Nerve, LLC, Lewes, DE) and an online survey. This investigation was determined to be exempt by the institutional review boards of NU and UC.

Historical data from scheduling software was available for all applicants. Survey questions were developed iteratively by a focus group of EM education experts with the goal of assessing applicants’ attitudes and experiences while interviewing in Chicago. This survey contained both multiple-choice and free-text items. Respondents could choose to skip any questions. For numerical calculations involving open-ended responses (e.g. self-reported cost of interviews), we excluded non-numerical responses (e.g. “low”). Unanswered questions were treated as null.

The survey was distributed electronically in March and April 2016 to all residents invited to interview at both programs. Responses were collected through May 2016. All applicants had valid e-mail addresses on file. Survey completion was optional with no consequences for non-completion. Opening the survey from the informational e-mail was treated as consent to participate.

The target audience for this intervention was applicants from medical schools outside Illinois who completed interviews at both programs. In-state applicants were used as a proxy for Chicago-area applicants since asking for medical schools may have led to individually-identifiable data and only three applicants receiving both interviews attended in-state medical schools with campuses outside the Chicago area. We used data from interview scheduling software to identify this group and a subgroup of applicants who interviewed at both programs in the same calendar week. We extrapolated the average cost of completing interviews at both programs using inflation-adjusted travel and lodging cost estimates adopted from a 2008 study by Kerfoot et al. ($225 for travel, $130 for food and lodging per interview).^3^ We conducted data compilation and analysis using Microsoft Excel (Microsoft, Redmond, WA).

## RESULTS

**Figure 1:**
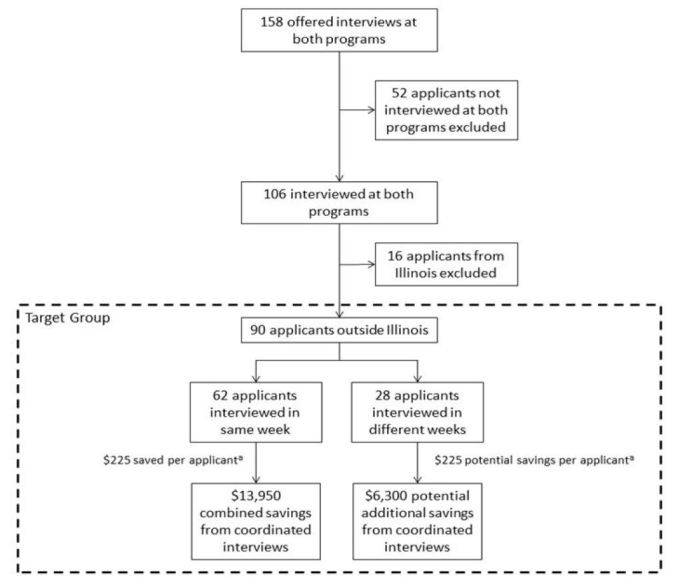
Applicant characteristics and estimated costs of interviewing. ^a^Standard cost assumptions: $225 travel, $130 per night food and lodging.^2^ $485 for same-week applicants (standard travel costs, 2 x food and lodging costs). $710 for separate-week applicants (2 x travel costs, 2 x food and lodging costs).

Historical data used to identify the target group and estimate cost savings of same-week interviews is outlined in Figure 1. The estimated total cost savings of coordinated interview dates was $13,950. An additional $6,300 in potential cost savings was identified in the subgroup of target applicants who did not complete both interviews in the same week.

The overall response rate for the survey was 53% (84 of 158 applicants invited to both programs). We received 45 responses out of the 90 target group applicants (50% subgroup response rate). Non-target group responses included the following independent exclusion criteria: 11 respondents interviewed at NU only, four interviewed at UC only, four neither interviewed at NU nor UC, 13 attended medical school in Illinois, and 13 did not indicate the location of their medical school.

The majority of target group respondents made only one trip to Chicago (51%, 23 of 45), whereas 33% (15 of 45) made two trips and 16% (7 of 45) reported making three or more trips. Most target group respondents were able to schedule both interviews in the same week (67%, 30 of 45). Of target respondents who completed both interviews in the same week, 67% (20 of 30) were either unsure or confident they would not have made a second trip to Chicago if coordinated interviews were not available.

Only 30% (13 of 44) of target group respondents reported awareness of the intervention; however, these respondents were not more likely to schedule a same-week interview (69% same-week interviews in respondents aware of the intervention, 68% same-week interviews in respondents unaware of the intervention). The mean self-estimated cost per trip to Chicago was $380 (standard deviation: $236) for target group respondents, which was near our literature-derived estimate of $355 per trip.

**Figure 2:**
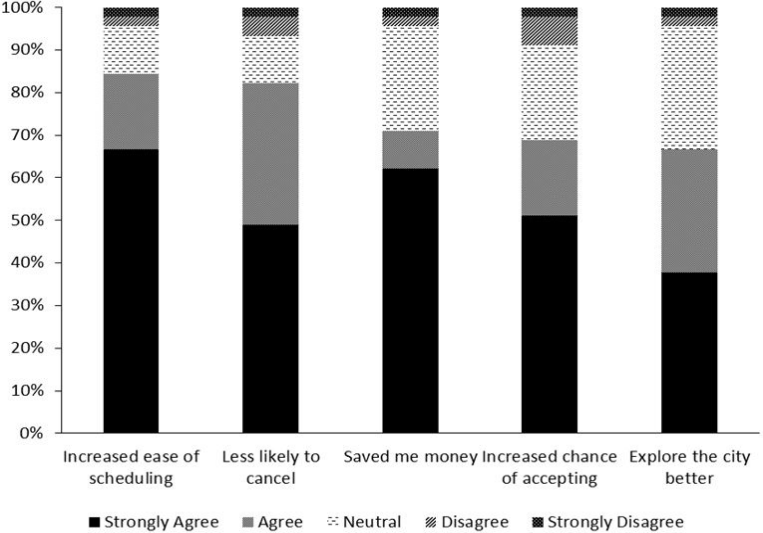
Target group attitudes regarding improvement in the interview experience from coordinated interviews of applicants to two emergency medicine residency programs in Chicago.

Survey items that evaluated target group satisfaction with this intervention are illustrated in Figure 2. All 45 applicants from the target group responded to each question. Most applicants reported a positive impact of the intervention in all measured categories.

## DISCUSSION

This is the first report of an intervention designed to reduce the costs and burden of travel for residency applicants interviewing at different programs in the same city. With minimal administrative effort from the coordinating programs, we were able to create a schedule that was associated with significant estimated cost savings and that applicants viewed favorably in every measured category.

In the future we hope to further increase the proportion of residents scheduling interviews in the same week by making a deliberate effort to advertise this intervention and its potential benefits. Additionally, we believe that some applicants who wished to schedule consecutive interviews were unable to do so due to lack of availability. We plan to support fair access to all dates by sending an e-mail with an invitation to schedule interviews at a specified time the next day.

Another potential confounder is that our metropolitan area contains several other EM residency programs. It is possible that applicants who interviewed at our programs on different weeks coordinated interviews with other programs in Chicago. This phenomenon would support our findings of convenience and cost-savings through coordination of interviews; however, this impact would not be captured in our study. While involving more residency programs in this intervention could create and capture increased savings and convenience for applicants, the magnitude of this effect would be attenuated by the degree of overlap in applicant pools. In addition, a high pre-existing overlap in applicant pools should help guard against an artificial narrowing of the field of applicants interviewing in the area. We found a high degree of overlap in our respective applicant pools (over 150 shared invitations the year of the intervention). A logical first step in broadening or recreating this intervention would be to estimate potential impact by assessing the degree of applicant pool overlap between participating institutions.

While the measures of applicant benefit from coordinated interviews are promising, interpretation of potential benefit to the program is more nuanced. Among applicants who interviewed at both institutions, over half of respondents stated that they were unsure or would not have made a second trip to Chicago if coordinated interview days were not available. This suggests that coordinated interview dates attract applicants who might not otherwise have interviewed. Whether this is truly a desirable outcome is questionable, as candidates who interview at a program out of convenience may be less interested and less likely to rank the program highly. In this hypothetical, the interview spot might be better used for another applicant with greater interest in the program. However, the authors’ opinion is that the opportunity for increased exposure to highly competitive applicants representing broad geographical diversity outweighs the risk of interviewing applicants with lower initial interest in the program.

This intervention could have a considerable impact at scale. Per the Society for Academic Emergency Medicine Residency Directory, 20 U.S. cities are home to two or more EM residencies. By coordinating interview dates within these cities, as many as 67 (36.7%) programs could benefit their applicants with this intervention. This effect could be greater still if expanded to other specialties.

## LIMITATIONS

Our survey had a 53% response rate, which may lead to non-response bias. However, given the lack of a perceived negative impact and the minimal time investment required for deployment, we believe that this intervention is worth pursuing for the sake of those demonstrated to benefit, even if the remainder of responses would not have indicated a benefit.

In addition, many factors contribute to costs of interviewing and applicant recall of costs may be inaccurate. Our pre-determined cost estimates closely resembled the mean cost reported by applicants ($355 vs. $380, respectively). The close relation of these variables contributes validity evidence to the estimates in this study, notwithstanding the complex nature of the variables involved.

## CONCLUSION

Applicants favorably viewed the coordinated scheduling of interview dates between nearby residency programs across all measures of experience. Increased efforts to improve availability of coordinated interviews may lead to greater cost reductions for applicants.
